# The outcomes of bariatric surgery on rheumatoid arthritis disease activity: a prospective cohort study

**DOI:** 10.1038/s41598-020-59723-8

**Published:** 2020-02-21

**Authors:** Fang Xu, Chao Yu, De-Guan Li, Qiang Yan, Shang-Xin Zhang, Xiao-Dong Yang, Zhen Zhang

**Affiliations:** Department of Gastrointestinal Surgery, First Affiliated Hospital of Anhui Medical University, Hefei, 230022 China

**Keywords:** Stomach, Rheumatoid arthritis

## Abstract

Rheumatoid arthritis (RA) is a chronic inflammatory autoimmune disease that primarily affects the joints. Overweight and obesity can aggravate disease activity and clinical outcome in patients with RA. However, the role of bariatric surgery in inducing weight loss in the treatment of RA has not been confirmed. In this 12-month prospective cohort study, RA patients with obesity who were referred to our hospital were included. Thirty-two patients were classified into the bariatric surgery group according to the patient’s decision after a comprehensive assessment of surgery indications, and 33 patients received only pharmacotherapy for RA. At the 12-month follow-up, the response rates of ACR20, ACR50 and ACR70 were 75.0% vs. 51.5%, 53.1% vs. 39.4% and 31.3% vs. 21.2% in the bariatric surgery and non-surgery groups, respectively (all p < 0.05); the mean DAS28-ESR, DAS28-CRP and cDAI scores were 1.5 ± 0.9 vs. 2.4 ± 1.4, 1.2 ± 0.9 vs. 2.2 ± 1.7 and 9.5 ± 6.8 vs. 15.8 ± 12.5, respectively, in surgical patients compared to non-surgical patients (all p < 0.05). Compared to baseline, after 12 months, a significant reduction was observed in the use of leflunomide, biological agents, combination treatments, and NSAIDs in both groups (p < 0.05 or p < 0.01). However, there was no difference in medication use between the 2 groups either at baseline or at the 12-month follow-up (all p > 0.05). Compared to non-surgical patients, in RA patients with obesity, weight loss after bariatric surgery was associated with lower disease activity. Medication tapering for RA in patients who underwent bariatric surgery was not superior to that in non-surgical patients.

## Introduction

Rheumatoid arthritis (RA) is a chronic inflammatory autoimmune disease that primarily affects the joints and results in severe pain and swelling, progressive damage and disability, which ultimately lead to physical malfunction and even early death^1,2^. Although there is a complicated pathogenesis for RA, several factors have been identified that represent the possible causes for the onset and development of RA, including female sex, genetic predisposition and socioeconomic status^3^. Recent studies have demonstrated that an increase in body mass index contributes to a higher risk of developing RA^4–6^.

The global prevalence of obesity and associated diseases has increased considerably in recent decades^7^. Overweight and obesity can aggravate disease activity and clinical outcomes via changing histological features and the inflammatory gene signature of the synovial membrane in patients with RA, especially among female patients^8^. Furthermore, RA patients generally take immunosuppressive therapy, which has numerous adverse effects, including susceptibility to infection and weight gain^9^. RA patients with obesity have a worse response to therapy, including treatment with conventional synthetic disease modifying antirheumatic drugs (csDMARDs), such as methotrexate, sulfasalazine, hydroxychloroquine and leflunomide, biologic DMARDs (bDMARDs), including tumour necrosis factor-α inhibitors and non-TNFα inhibitors, or targeted synthetic DMARDs (tsDMARDs)^10,11^. Assessment and improvement of weight indicators should be an important part of the routine care of RA patients in clinical practice^12^.

Weight loss through lifestyle and dietary modification has been shown to improve the clinical manifestations of RA^13^. After substantial weight loss from bariatric surgery, RA patients had further decreased disease activity, lower serum inflammatory markers, less RA-related medication use, and lower RA cause-specific mortality^14^. However, other factors, such as improved physical activity and metabolic changes after bariatric surgery, may also have contributed to these postsurgical improvements^14^. On the other hand, studies have demonstrated that weight loss is also associated with worsening disability, possibly due to its association with chronic illness and the development of age-related or disease-related frailty^15,16^. However, the role of weight loss in the treatment of RA has not been confirmed since the efficacy has not been studied in a controlled trial with a comparison group. This prompted us to conduct an investigator-initiated, 12-month study to determine whether bariatric surgery is effective in improving the clinical outcomes in active RA patients with obesity compared with similar RA patients who did not undergo bariatric surgery. This study also evaluated other aspects of the RA-related medication tapering profile of bariatric surgery in RA patients with obesity.

## Results

### Patient enrolment

**Figure 1:**
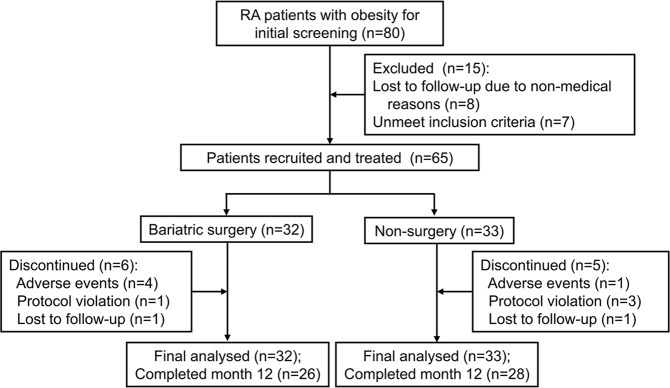
Study profile and patient disposition.

All 80 RA patients with obesity were initially screened, and 65 eligible patients were enrolled in the study. According to the patient’s decision and surgery indications, 32 patients underwent bariatric surgery for obesity and received pharmacotherapy for RA, and 33 patients received only pharmacotherapy. In the bariatric surgery group, 6 patients discontinued treatment before week 12 because of adverse events, and 1 patient discontinued treatment due to a protocol violation and was lost to follow-up. In the non-surgery group, 5 patients discontinued treatment due to adverse events (n = 1), protocol violation (n = 3), or loss to follow-up (n = 1) before the final evaluation at week 12. Finally, 81.3% (26/32) and 84.8% (28/33) of patients in the bariatric surgery and non-surgery groups, respectively, completed the 12-month evaluation. The study profile and patient disposition are shown in Fig. 1.

### Clinical characteristics

In the bariatric surgery and non-surgery groups, the mean ages were 51.8 ± 11.8 years and 57.6 ± 16.4 years, respectively, which were significantly different (p = 0.04); 90.6% and 91.0% of patients were women; 12.5% and 15.2% had smoking habits; the RA durations were 10.3 ± 4.8 years and 8.5 ± 4.9 years; and body weights were 111.8 ± 11.8 kg and 108.5 ± 9.5 kg, respectively. All patients had active disease, which was reflected by the number of tender joints (TJCs) and swollen joints (SJCs), erythrocyte sedimentation rate (ESR) and C-reactive protein (CRP) values, and the PtGA, PyGA, and high DAS28 and cDAI scores. There were no statistically significant differences among the 2 groups for any of the aforementioned variables (p > 0.05) except for PtGA (p = 0.03). In the bariatric surgery group, the surgery type included Roux-en-Y gastric bypass 19/32 (59.4%) and laparoscopic sleeve gastrectomy 13/32 (40.6%). The comorbidities of the recruited patients included osteoarthritis (75.0% vs. 66.7%), hypertension (40.6% vs. 33.3%), type 2 diabetes (28.1% vs. 24.2%), congestive heart failure (12.5% vs. 6.1%), coronary artery disease (3.1% vs. 9.1%), and chronic obstructive pulmonary disease (6.3% vs. 6.1%); these variables were not significantly different between the bariatric surgery and non-surgery groups (p > 0.05). The baseline demographic and clinical characteristics of the included patients are detailed in Table 1.

**Table 1:** Baseline demographic and clinical characteristics of recruited patients.

Characteristics	Bariatric surgery (n = 32)	Non-surgery (n = 33)	P
Age (SD), years	51.8 (11.8)	57.6 (16.4)	0.04
Female, n (%)	29 (90.6)	30 (91.0)	0.97
Smoking, n (%)	4 (12.5)	5 (15.2)	0.76
RA duration (SD), years	10.3 (4.8)	8.5 (4.9)	0.15
Weight (SD), kg	111.8 (11.8)	108.5 (9.5)	0.22
Body mass index (SD), kg/m^2^	38.4 (14.8)	37.2 (12.5)	0.17
TJC (SD), n	14.3 (7.9)	13.2 (5.9)	0.12
SJC (SD), n	8.4 (6.2)	7.9 (4.1)	0.30
RF positive, n (%)	23 (71.9)	25 (75.8)	0.72
Anti-CCP positive, n (%)	24 (75.0)	27 (81.8)	0.50
ESR (SD), mm/h	40.7 (19.2)	37 (10.2)	0.13
CRP (SD), mg/L	46.8 (14.5)	45.3 (14.3)	0.80
PtGA (SD), mm	68.8 (13.7)	61.6 (18.5)	0.03
PyGA (SD), mm	67.2 (14.7)	61.2 (10.6)	0.06
DAS28-ESR (SD)	6.3 (1.4)	6.2 (1.0)	0.68
DAS28-CRP (SD)	6.6 (3.7)	5.8 (2.9)	0.36
cDAI (SD)	38.9 (10.8)	37.5 (11.8)	0.62
**Bariatric surgery type, n (%)**			
Roux-en-Y gastric bypass	19 (59.4)	—	—
Laparoscopic sleeve gastrectomy	13 (40.6)	—	—
**Comorbidities, n (%)**			
Osteoarthritis	24 (75.0)	22 (66.7)	0.46
Hypertension	13 (40.6)	11 (33.3)	0.54
Type 2 diabetes	9 (28.1)	8 (24.2)	0.72
Congestive heart failure	4 (12.5)	2 (6.1)	0.37
Coronary artery disease	1 (3.1)	3 (9.1)	0.32
Chronic obstructive pulmonary disease	2 (6.3)	2 (6.1)	0.96

RA, rheumatoid arthritis; SJC, swollen joint count; TJC, tender joint count; RF, rheumatoid factor; Anti-CCP, anti-cyclic citrullinated peptide antibody; ESR, erythrocyte sedimentation rate; CRP, C-reactive protein; PtGA, patient’s global assessment of overall well-being; PyGA, physician’s global assessment; DAS28, 28-joint count disease activity score; cDAI, clinical disease activity index.

### Treatment efficacy

In RA patients with obesity who underwent bariatric surgery, patients lost substantial weight after surgery: their mean weight was 111.8 ± 11.8 kg at baseline, 92.8 ± 19.3 kg at 4 months, 86.2 ± 23.7 kg at 8 months, and 78.2 ± 25.6 kg at 12 months (p < 0.05 or <0.01 for all time points compared to baseline). The body mass index (BMI) was 38.4 ± 4.8 kg/m^2^ at baseline, 31.9 ± 10.2 kg/m^2^ at 4 months, 29.6 ± 11.9 kg/m^2^ at 8 months, and 26.9 ± 13.5 kg/m^2^ at 12 months of follow-up (p < 0.05 or < 0.01 for all time points compared to baseline) with a corresponding reduction of 61.4 ± 32.3%, 75.2 ± 34.4% and 79.4 ± 39.6% excess weight loss (p < 0.01 for all time points compared to baseline). In non-surgical patients, however, the mean weight was 108.5 ± 9.5 kg at baseline, 106.7 ± 15.5 kg at 4 months, 104.5 ± 12.5 kg at 8 months, and 103.5 ± 17.5 kg at 12 months (p > 0.05 for all time points compared to baseline). Accordingly, the BMI was 37.2 ± 12.5 kg/m^2^, 36.6 ± 11.3 kg/m^2^, 35.8 ± 12.8 kg/m^2^, and 35.3 ± 13.2 kg/m^2^ at baseline and at 4, 8, and 12 months of follow-up, respectively (p > 0.05 for all time points compared to baseline) (Table 2).

**Table 2:** Anthropometric parameters of subjects over time after bariatric surgery.

Parameters	Baseline	Month 4	Month 8	Month 12
**Bariatric surgery, mean (SD)**				
Weight, kg	111.8 (11.8)	92.8 (19.3)**	86.2 (23.7)**	78.2 (25.6)**
Change in weight, kg	N/A	−19.5 (13.5)*	−25.1 (15.9)**	−33.0 (16.5)**
Excess weight loss, %	N/A	61.4 (32.3)**	75.2 (34.4)**	79.4 (39.6)**
BMI, kg/m^2^	38.4 (4.8)	31.9 (10.2)**	29.6 (11.9)**	26.9 (13.5)**
Change in BMI, kg/m^2^	N/A	−6.7 (3.5)*	−8.6 (5.9)**	−11.3 (6.7)**
**Non-surgery, mean ± SD**				
Weight, kg	108.5 (9.5)	106.7 (15.5)	104.5 (12.5)	103.5 (17.5)
Change in weight, kg	N/A	−2.2 (1.6)	−4.9 (3.5)	−5.1 (4.9)
Excess weight loss, %	N/A	8.5 (22.4)	9.1 (21.6)	11.2 (29.5)
BMI, kg/m^2^	37.2 (12.5)	36.6 (11.3)	35.8 (12.8)	35.3 (13.2)
Change in BMI, kg/m^2^	N/A	−1.4 (0.89)	−1.6 (1.1)	−1.7 (1.2)

*P < 0.05 and **P < 0.01 compared to baseline. N/A, not applicable; BMI, body mass index.

**Figure 2:**
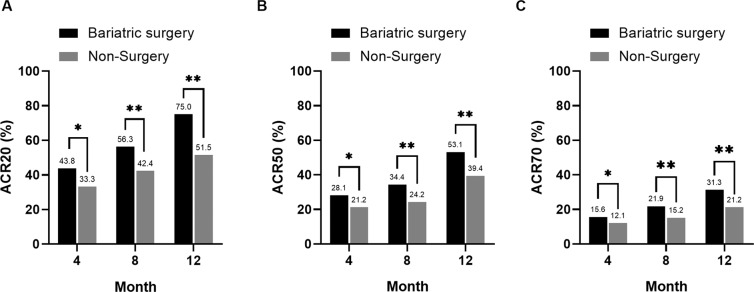
The efficacy measures over time in RA patients. *P < 0.05 and **P < 0.01 compared between 2 groups. ACR20/50/70, American College of Rheumatology 20/50/70% improvement criteria.

An ACR20 response rate was observed for 12/28 patients (43.8%) in the bariatric surgery group and for 11/33 patients (33.3%) in the non-surgery group at 4 months (p < 0.05); the ACR20 response was observed for 15/26 (56.3%) and 13/30 (42.4%) patients in the bariatric surgery and non-surgery groups, respectively, at 8 months (p < 0.01); the ACR20 response was observed for 20/26 (75.0%) and 14/28 (51.5%) patients in the bariatric surgery and non-surgery groups, respectively, at 12 months (p < 0.01). ACR50 responses were achieved by 28.1%, 34.4%, and 53.1% of RA patients who underwent bariatric surgery at 4, 8, and 12 months, respectively, which were significantly different from those in patients in the non-surgery group at each time point (21.2%, 24.2%, and 39.4%, respectively) (p < 0.05 or p < 0.01). ACR70 responses were achieved by 15.6%, 21.9%, and 31.3% for RA patients who underwent bariatric surgery at 4, 8, and 12 months, respectively, which were significantly different from those in patients in the non-surgery group (12.1%, 15.2%, and 21.2%, respectively) (p < 0.05 or p < 0.01) (Fig. 2).

**Figure 3:**
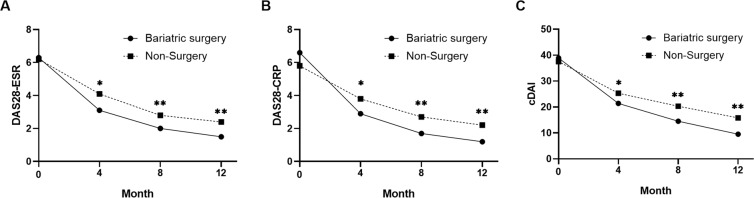
The parameters of disease activity over time in RA patients. *P < 0.05 and **P < 0.01 compared between 2 groups. DAS28-ESR, Disease Activity score in 28 joints based on erythrocyte sedimentation rate; DAS28-CRP, Disease Activity score in 28 joints based on C-reactive protein.

The mean DAS28-ESR scores were 6.3 ± 1.4, 3.1 ± 1.2, 2.0 ± 1.5, and 1.5 ± 0.9 in the surgical RA patients at baseline and at 4, 8, and 12 months of follow-up, respectively, which were significantly different from the scores in non-surgical patients at each time point (6.2 ± 1.0, 4.1 ± 1.3, 2.8 ± 1.1, and 2.4 ± 1.4, respectively) (p < 0.05 or p < 0.01). The mean DAS28-CRP scores were 6.6 ± 3.7, 2.9 ± 2.1, 1.7 ± 1.4, and 1.2 ± 0.9 in the surgical patients at baseline and at 4, 8, and 12 months of follow-up, respectively, which were significantly different from the scores for non-surgical patients at each time point (5.8 ± 2.9, 3.8 ± 1.2, 2.7 ± 1.8, and 2.2 ± 1.7, respectively) (p < 0.05 or p < 0.01). Finally, the mean cDAI scores were 38.9 ± 24.5, 21.4 ± 18.3, 14.5 ± 8.9, and 9.5 ± 6.8 at baseline and at 4, 8, and 12 months of follow-up in surgical patients, respectively, which were also significantly different from the scores in the non-surgery group (37.5 ± 21.0, 25.3 ± 17.5, 20.3 ± 14.2 and 15.8 ± 12.5, respectively) (p < 0.05 or p < 0.01) at each time point (Fig. 3).

### Medication tapering

At baseline, the patients’ medication for RA included csDMARDs in 31/32 patients (96.9%), biological agents in 14/32 patients (43.8%), combination treatment including 2 or more immunosuppressive medications in 21/32 patients (65.6%), glucocorticoids in 4/32 patients (12.5%), and NSAIDs in 16/32 patients (50.0%) in the bariatric surgery group. The patients’ medication for RA in the non-surgical group included csDMARDs, biological agents, combination treatment, glucocorticoids, and NSAIDs in 31/33 (93.9%), 10/33 (30.3%), 19/33 (57.6%), 5/33 (15.2%), and 17/33 patients (51.5%), respectively. There were no significant differences between the 2 groups (all p > 0.05). Compared to baseline, after 12 months, there was a significant reduction in the use of leflunomide, biological agents, combination treatment, and NSAIDs in both groups (p < 0.05 or p < 0.01). However, medication use did not differ between the 2 groups at baseline or at the 12-month follow-up (all p > 0.05) (Table 3). Medication tapering for RA patients who underwent bariatric surgery was, therefore, not superior to that in non-surgical patients.

**Table 3:** Patients’ medication at baseline and 12 months of follow-up.

Medication	Baseline, n (%)			End follow-up, n (%)		
	Bariatric surgery (n = 32)	Non-surgery (n = 33)	P	Bariatric surgery (n = 26)	Non-surgery (n = 28)	P
csDMARDs	31 (96.9)	31 (93.9)	1.00	23 (88.5)	21 (75.0)	0.30
Methotrexate	28 (87.5)	27 (81.8)	0.77	19 (59.4)	20 (71.4)	0.89
Hydroxychloroquine	15 (46.9)	12 (36.4)	0.39	10 (31.3)	9 (32.1)	0.63
Leflunomide	18 (56.3)	15 (45.5)	0.38	5 (19.2)**	5 (17.9)*	1.00
Sulfasalazine	5 (15.6)	4 (12.1)	0.96	3 (9.4)	2 (7.1)	0.93
Biological agents^†^	14 (43.8)	10 (30.3)	0.39	5 (15.6)*	6 (21.4)	0.84
Combination^‡^	21 (65.6)	19 (57.6)	0.51	9 (28.1)*	8 (28.6)*	0.63
Glucocorticoids	4 (12.5)	5 (15.2)	1.00	0 (0)	1 (3.6)	1.00
NSAIDs	16 (50.0)	17 (51.5)	1.00	2 (6.3)**	7 (25.0)*	0.14

*P < 0.05 and **P < 0.01 compared between patients in the same group at the 12-month follow-up and baseline.

^†^Biological agents including TNFα inhibitors or JAK inhibitors. ^‡^Combination treatment including 2 or more immunosuppressive medications.

csDMARDs, conventional synthetic disease modifying antirheumatic drugs; NSAIDs, nonsteroidal anti-inflammatory drugs.

### Postoperative complications

In RA patients who underwent bariatric surgery, early postoperative complications within 1 month were observed in 5/32 (15.6%) patients, including anastomotic ulcer/leak (n = 3), intra-abdominal hernia (n = 1), and postoperative gastric functional emptying (n = 1). Late complications more than 1 month after surgery were observed in 3/32 (9.4%) patients, including an incisional hernia (n = 1), intestinal obstructions (n = 1) and serious wound infection (n = 1). In this study, no patients who underwent bariatric surgery had more than one postoperative complication.

## Discussion

Obesity, with a higher prevalence, is one of the described risk factors for the onset of RA, and it negatively affects disease activity and treatment outcomes^4,17^. The benefits of bariatric surgery beyond the weight loss effect on disease and prognosis continue to be reported in other autoimmune or inflammatory diseases, including systemic lupus erythaematosus, inflammatory bowel disease, autoimmune thyroiditis and autoimmune liver diseases^18–21^. The association between obesity and RA might present a strategy for the prevention or control of RA activity^22,23^. Although it has been shown that substantial weight loss from bariatric surgery results in lower disease activity and improved response to treatment^14^, the effect of bariatric surgery on the treatment of RA was unconfirmed in a prior cohort study. Our study demonstrated that compared with non-surgical patients who were followed for up to 12 months, RA patients with obesity who underwent bariatric surgery had significant weight loss and a better response to treatment. RA-related medication usage, on the other hand, had no difference between the surgical and non-surgical patients at baseline or at the 12-month follow-up.

However, studies from Baker *et al*.^15,16^ showed that both severe obesity and weight loss were associated with worsening disability, and weight loss was a risk factor for death in patients with RA. Mounting studies have shown that obesity augments RA disease activity and decreases the likelihood of achieving sustained remission despite intensive treatment^24,25^. Non-surgical weight loss interventions contributed to a weight loss of 4.5 kg and significantly improved physical function in 19 overweight RA patients^26^. Furthermore, a more substantial strategy for weight loss, such as surgery, also has an impact on the levels of various inflammatory markers induced by obesity^27–30^. Santos *et al*.^31^ enrolled 46 patients with obesity-related metabolic dysfunction and showed that gastric banding surgery resulted in a significant decrease in the inflammation process associated with adipose tissue loss. Interestingly, in a Swedish study followed up for 29 years, there was no association between bariatric surgery and the incidence of RA in obese patients^32^, which might demonstrate that bariatric surgery has no preventive effect on the occurrence of RA.

Few prior studies have specifically focused on the effects of more substantial weight loss on RA severity before and after bariatric surgery. To the authors’ knowledge, the only report is a retrospective cohort study^14^ of bariatric surgery in 53 RA patients with moderate to high RA disease and a BMI of 47.8 ± 7.7 kg/m^2^. This study concluded that bariatric surgery resulted in a significant disease remission rate of 74% at a follow-up of 5.8 ± 3.2 years after surgery compared to a rate of 26% at baseline^14^. This study design was an uncontrolled cohort without a control group. It is therefore unclear whether the observed improvement in RA activity after bariatric surgery is related to RA-related medications or to surgery-specific effects. In the present study, both surgical and non-surgical patients had significantly decreased disease activity at 12 months compared to baseline, which was consistent with the abovementioned study. More importantly, after 12 months of follow-up, compared with the non-surgery group, the surgical patients also had a better response rate to RA-related medication in terms of ACRs, DAS28, and cDAI scores, providing powerful evidence to support the potential role of bariatric surgery in the treatment of RA.

The study of bariatric surgery’s efficacy on 31 patients with systemic lupus erythaematosus demonstrated that 42% of patients showed a reduction in the number of immunosuppressive medications and that 19.3% were off steroids completely at a mean follow-up of 3 years^33^. These findings suggest that surgery-induced weight loss is associated with decreased SLE immunosuppression medication requirements^33^. The study has the same issue that, due to the lack of comparison group data, it is uncertain whether the medication reduction is attributable to bariatric surgery or SLE-related treatment. A study of bariatric surgery in RA patients showed a significantly decreased usage of RA-related medication after bariatric surgery (66% at 1 year after surgery compared to 98% at baseline)^14^. In our study, there was a significant reduction in the use of leflunomide, biological agents, combination treatment, and NSAIDs in both surgical and non-surgical patients after 12 months compared to the baseline (p < 0.05 or p < 0.01). However, there were no differences in medication use between the 2 groups either at baseline or after 12 months of follow-up. Our data suggested that medication tapering for RA patients who underwent bariatric surgery was not superior to that in non-surgical patients.

There are some limitations to our study. First, the study was an open-label clinical study, and the treating doctors and patients were not blinded to the therapeutic strategy. Even so, the disease activity measurements were performed by different trained evaluators who were unaware of the specific therapeutic regimen to make the study more objective. Second, previous studies demonstrated that metabolic factors, including dietary intake and physical activity, were associated with the occurrence and disease activity^34–36^. However, as the setup of the parameters was mainly focused on the change in RA disease activity before and after treatment, data on these metabolic factors were not collected in this study. The deficiency of these metabolic factors might confound the treatment efficacy of bariatric surgery on RA disease activity. Finally, other limitations, including a lower case number, nonrandomized design, and short-term follow-up, need to be perfected in future studies.

To the best of our knowledge, this is the first cohort study to evaluate the treatment efficacy of bariatric surgery on RA patients with obesity compared with non-surgical patients. Our data demonstrated that bariatric surgery appears to be feasible in RA patients with obesity. The activity-improving effects of bariatric surgery were evident 4 months after surgery and persisted for at least 12 months. In conclusion, the present results suggest that weight loss may have an important role in the nonpharmacologic management of RA patients. These findings warrant further study concerning the treatment of RA patients, especially those with obesity.

## Methods

### Design overview

Approval for this retrospective study was granted by the ethics review board of the First Affiliated Hospital of Anhui Medical University (AHEC2013-015), and all methods were performed in accordance with the relevant ethical guidelines and regulations. Prior written informed consent was obtained from all patients. This 12-month cohort study was conducted between May 2016 and August 2018. RA patients with obesity who were referred to our hospital for surgical treatment of obesity and pharmacotherapy for RA were included.

### Participants and interventions

The eligible patients for this study had to meet the following criteria: (1) RA diagnosis determined by 2010 ACR/EULAR classification criteria^37^; (2) age of 18–65 years; (3) BMI ≥30 kg/m^2^ in 3 continuous years; (4) no abuse of alcohol or psychotropic drugs, and no serious behaviour disorder or mental retardation; (5) TJCs ≥5 and SJCs ≥3; and (6) ESR ≥20 mm/h or CRP ≥20 mg/L. Patients were excluded if they had (1) a history or current infection or any type of malignant cancer; (2) secondary obesity, such as Cushing syndrome, hypothyroidism, polycystic ovary syndrome, hypopituitarism, hypothalamic obesity, or prolactinoma; and (3) intolerance to surgery due to serious organ diseases. During the study, nonsteroidal anti-inflammatory drugs (NSAIDs) and low-dose glucocorticoids of ≤50 mg prednisone or equivalent/day were allowed when necessary.

All patients with RA were classified into either a bariatric surgery group or a non-surgery group after the comprehensive assessment of surgery indications according to the patient’s decision. Bariatric surgery was defined as Roux-en-Y gastric bypass (LRYGB) or laparoscopic sleeve gastrectomy (LSG). The RA-related treatment regimen was determined by rheumatologists. The disease activity measurements were performed by different trained evaluators who were unaware of the specific therapeutic regimen.

### Data collection

Data collected included baseline demographic characteristics, clinical parameters, operative data, RA activity index scores, and postoperative and follow-up outcomes. The demographic characteristics of the recruited patients included age, sex, height, weight, BMI, comorbidities, and the disease duration of RA. The clinical parameters included rheumatoid factor (RF), anti-cyclic citrullinated peptide antibody (Anti-CCP), ESR, and CRP. Operative data included surgery type and time, estimated blood loss, and intraoperative complications. The RA activity index included the patient’s global assessment of overall well-being (PtGA), the physician’s global assessment (PyGA) at baseline, the 28-joint count disease activity score (DAS28), and the clinical disease activity index (cDAI) at baseline and at the 4-, 8-, and 12-month follow-up. The postoperative and follow-up outcomes assessed were the percentage of patients who achieved the American College of Rheumatology criteria (ACR20, ACR50, and ACR70 represent ≥20%, 50% and 70% improvement, respectively), weight loss and excess weight loss (percentage of baseline weight above BMI 25 kg/m^2^ lost)^14^ at the 4-, 8-, and 12-month follow-up, and complications and changes in immunosuppressive medication at the 12-month follow-up.

### Statistical analysis

Data are presented as the mean and standard deviation (SD) for measurement variables and frequency percentages for categorical variables. Parameters were analysed using the unpaired Student’s *t* test (or the Mann-Whitney U test) for measurement variables and the *χ*^2^ test (or Fisher’s exact test) for categorical variables. Covariance analysis was used to compare the difference in disease activity over time in surgical and non-surgical patients. Patients with missing end points were considered to be nonresponders. Values of p < 0.05 were considered statistically significant. Statistical analysis and plotting were performed using SPSS 23 (IBM Corporation, Armonk, New York, NY, United States) and GraphPad Prism 7 (GraphPad, San Diego, CA, United States).

### Institutional review board statement

The study was reviewed and approved by the Medical Science Ethics Committee of First Affiliated Hospital of Anhui Medical University.

### Informed consent statement

All study participants, or their legal guardian, provided informed written consent prior to study enrollment.
